# Genetic variation analysis of the cosmopolitan chaetognath *Sagitta enflata* in the northern South China Sea based on mitochondrial COI gene sequences

**DOI:** 10.1080/23802359.2018.1535839

**Published:** 2018-11-21

**Authors:** Shina Wei, Min Yang, Yuan Dong, Qiwei Qin

**Affiliations:** a College of Marine Sciences, South China Agricultural University, Guangzhou, China;; b State Key Laboratory of Tropical Oceanography, South China Sea Institute of Oceanology Chinese Academy of Sciences, Guangzhou, China

**Keywords:** *Sagitta enflata*, Mitochondrial DNA, Cytochrome c oxidase subunit I, Genetic diversity

## Abstract

In this study, genetic diversity and population genetic structure of *Sagitta enflata* in the northern South China Sea were investigated by 623 bp fragment of mtDNA COI gene sequence. A total of 146 individuals were collected from nine stations and 92 different haplotypes were obtained. 485 variable sites (210 were parsimony informative and 275 were singleton variable sites), and no insertion or deletion was found. An analysis of molecular variance (AMOVA) and conventional population statistics (*F*_ST_) revealed a low level of genetic differentiation among nine populations (*F*_ST_ = 0.14794, *p* < .05), indicating no geographical patterning among nine populations. The present results were able to provide a reference for the phylogenetic relationships and assessment of the genetic structure of *S. enflata* in the northern South China Sea.

## Introduction

Chaetognaths (arrow worms) are small-sized and important predators in the marine ecosystem (Feigenbaum 1979), and copepods are the dominant prey of chaetognaths (Jennings et al. 2010). They can be found in coastal areas and in oceanic waters, from polar to tropical areas, and from the surface to several thousand meters depth (Alvarino 1965). The origins of the arrow worms remain obscure, but molecular studies will finally bring the true evolutionary relationship (Telford 2004). Taken as a whole, the genetic diversity of marine species is believed to present high levels within population and low levels between populations (Meriam et al. 2015). Many physical factors, such as climate, ocean currents, and lack of barriers in the open sea may explain this diversity (Maltagliati et al. 2002, 2010; Fernández et al. 2011).

*Sagitta* is a genus of holoplanktonic chaetognaths with about 70 species identified from the oceans around the world. These species numerically dominate mesozooplankton and are important secondary consumers in the pelagic ecosystem (Pierrot-Bults 1982). *Sagitta enflata* is a cosmopolitan epiplanktonic species in temperate coastal waters, tropical-subtropical epipelagic waters, and tropical-subtropical mesopelagic waters and occurs mainly in the upper 300 m (Øresland 2000; Tse et al. 2007). While several investigators have studied their wide geographic distribution and migration behavior, the evaluation of its genetic diversity and population genetic structure is also very important and has not been reported so far.

Mitochondrial DNA (mtDNA) is widely being used for elucidating molecular systematics studies. Moreover, cytochrome oxidase subunit I (COI) is considered as more rapid evolutionary rate and polymorphic than other mitochondrial genes, and therefore frequently used to study population genetic structure analysis and phylogeographic relationships of marine species (Rabaoui et al. 2011). In this study, we used sequences of the COI gene to investigate the genetic diversity and population variation of 146 *S. enflata* individual among nine populations from the different geographical distribution of northern South China Sea. The primary specimens have been deposited in the South China Sea Institute of Oceanology, Chinese Academy of Sciences, Guangzhou, China, and the accession number is SCSMBC040498. The results would be helpful for phylogenetic reconstructions and population genetic structure of this species.

## Materials and methods

### Sample collection and DNA extraction

**Figure 1.**
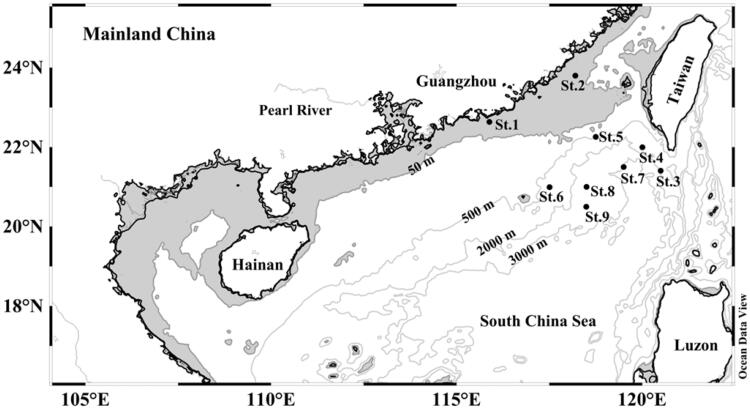
Location of sampling stations for *S. enflata* samples from nine populations in northern South China Sea (nSCS).

We collected 146 *S. enflata* individuals at nine stations in the northern South China Sea (Figure 1) during August 2015. Each individual was preserved in 95% ethanol before genomic DNA (gDNA) extraction. Total gDNA was extracted using marine animals gDNA kit (Biomiga, GD3311-02) and was visualized on 1.0% agarose gel to verify the quality of high molecular weight DNA extractions. Fragments of mitochondrial COI from all individuals of *S. enflata* were amplified using the primer pairs LCO1490 (5′-GGTCAACAAATCATAAAGATATTGG-3′) and HCO2198(5′-TAAACTTCAGGGTGACCAAAAAATCA-3′) (Folmer et al. 1994). Amplifications were performed in a total volume of 50 μL containing 10 × PCR buffer, 2.5 mM dNTP Mix, 10 μM each primer, 100 ng templates, and 2 U *Taq* DNA polymerase (Takara, Dalian, China). The PCR program was carried out under the following conditions: an initial denaturing at 94 °C for 4 min, followed by 30 cycles of denaturing at 94°C for 30 s, annealing at 50 °C for 50 s, and extension at 72 °C for 1 min, with a final extension at 72 °C for 10 min. The PCR products were visualized on 1% agarose gels, and purified with a Takara Agarose Gel DNA Purification Kit (Takara, China). Gene sequencing was performed on ABI 3730XL DNA Analyzer (Applied Biosystems, Foster City, CA).

### Data analyses

Both sequences (forward and reverse) of the COI gene fragment obtained for each specimen were aligned and edited by visual inspection using SeqScape version 2.5 (Applied Biosystems). Final alignments were optimized using BioEdit version 7.0.4.1 using *S. enflata* COI sequences as reference (GenBank accession no. KF977332). Nucleotide composition and variable sites were analyzed using MEGA3.1 (Kumar et al. 2004). Population structure of *S. enflata* was investigated using analysis of molecular variance (AMOVA), and the *F*_ST_ was examined using the Mantel test with 1000 permutations, performed by Arlequin version 3.01 (Excoffier et al. 2007).

## Results and discussion

Molecular genetic studies have shown the existence of genetic differentiation corresponding to different water masses or biogeographic boundaries in several zooplankton species (Bucklin et al. 2000; Goetze 2005). The investigations on chaetognaths were performed on some widespread species like *Parasagitta elegans*, *Parasagitta setosa*, *Caecosagitta macrocephala*, *Eukrohnia hamata* (Peijnenburg et al. 2005, 2006; Miyamoto et al. 2010; Kulagin et al. 2014). In this study, amplification of a 623bp fragment of the COI gene from 146 *S. enflata* individuals yielded 92 distinct haplotypes (GenBank accession nos. KX009784–KX009875). Among all 92 haplotypes, there were 485 variable sites (210 were parsimony informative and 275 were singleton variable sites), and no insertion or deletion was found. The mean total nucleotide composition was 22.4% A, 19.3% G, 23.0% C, and 35.3% T.

Compared to the previous reported studies on zooplankton, our estimates of genetic diversity within the *S. enflata* species are close to estimates of genetic diversity within species from other taxa. The analysis of molecular variance (AMOVA) is one of the most widely used methods of genetic data analysis (Excoffier et al. 1992). AMOVA indicated that 15.69% of variation was attributed to distribution among populations within groups and 85.21% to the distribution within populations (Table 1). Our results showed that the genetic diversity of *S. enflata* for the present study region included continental shelf, continental slope, and deep-sea basins, it may be explained by the Kuroshio current intrusion through the Luzon Strait into the nSCS. In order to further study the genetic structure of this worldwide species, more molecular markers and populations will be needed in a comprehensive analysis.

**Table 1.** AMOVA analysis of mtDNA COI gene sequences in nine populations of *S. enflata*.

Source of variation	df	Sum of squares	Variance components	Percentage of variation
Among groups	2	105.834	−0.14381Va	−0.9
Among populations within groups	6	309.915	2.50784Vb	15.69
Within populations	137	1865.278	13.61517Vc	85.21
Total	145	2281.027	15.9792	100

*F*_st_ = 0.14794 (*p* < .05).

## References

[CIT0001] Alvarino A 1965 Chaetognaths. Oceanogr Mar Biol Annu Rev. 3:115–194.

[CIT0002] Bucklin A, Astthorsson OS, Gislason A, Allen LD, Smolenack SB, Wiebe PH 2000 Population genetic variation of *Calanus finmarchicus* in Icelandic waters: preliminary evidence of genetic differences between Atlantic and Arctic populations. ICES J Mar Sci. 57:1592–1604.

[CIT0003] Excoffier L, Smouse PE, Quattro JM 1992 Analysis of molecular variance inferred from metric distances among DNA haplotypes: application to human mitochondrial DNA restriction data. Genetics. 131:479–491. 1644282 10.1093/genetics/131.2.479 PMC1205020

[CIT0004] Excoffier L, Laval G, Schneider S 2007 Arlequin (version 3.0): an integrated software package for population genetics data analysis. Evol Bioinform Online. 1:47–50. 19325852 PMC2658868

[CIT0005] Feigenbaum D 1979 Daily ration and specific daily ration of the chaetognath *Sagitta enflata*. Mar Biol. 54:75–82.

[CIT0006] Fernández MV, Heras S, Maltagliati F, Turco A, Roldán MI 2011 Genetic structure in the blue and red shrimp, *Aristeus antennatus* and the role played by hydrographical and oceanographical barriers. Mar Ecol Prog Ser. 421:163–171.

[CIT0007] Folmer O, Black M, Hoeh W, Lutz R, Vrijenhoek R 1994 DNA primers for amplification of mitochondrial cytochrome c oxidase subunit I from diverse metazoan invertebrates. Mol Mar Biol Biotechnol. 3:294–299. 7881515

[CIT0008] Goetze E 2005 Global population genetic structure and biogeography of the oceanic copepods *Eucalanus hyalinus* and *E. spinifer*. Evolution. 59:2378–2398. 16396179

[CIT0010] Jennings RM, Bucklin A, Pierrot-Bults A 2010 Barcoding of arrow worms (Phylum Chaetognatha) from three oceans: genetic diversity and evolution within an enigmatic phylum. PLoS One. 5:e9949. 20376348 10.1371/journal.pone.0009949 PMC2848590

[CIT0011] Kulagin DN, Stupnikova AN, Neretina TV, Mugue NS 2014 Spatial genetic heterogeneity of the cosmopolitan chaetognath *Eukrohnia hamata* (Möbius, 1875) revealed by mitochondrial DNA. Hydrobiologia. 721:197–207.

[CIT0012] Kumar S, Tamura K, Nei M 2004 MEGA3: integrated software for molecular evolutionary genetics analysis and sequence alignment. Brief Bioinformatics. 5:150–163. 15260895 10.1093/bib/5.2.150

[CIT0013] Maltagliati F, Belcari P, Casu D, Casu M, Sartor P, Vargiu G, Castelli A 2002 Allozyme genetic variability and gene flow in *Octopus vulgaris* (Cephalopoda, Octopodidae) from the Mediterranean Sea. Bull Mar Sci. 71:473–486.

[CIT0014] Maltagliati F, Giuseppe GD, Barbieri M, Castelli A, Dini F 2010 Phylogeography and genetic structure of the edible sea urchin *Paracentrotus lividus* (Echinodermata, Echinoidea) inferred from the mitochondrial cytochrome b gene. Biol J Linn Soc. 100:910–923.

[CIT0015] Meriam T, Wafa T, Khawla T, Tarek H, Abdeljelil G, Mhamed E 2015 Genetic diversity and population structure of *Sepia officinalis* from the Tunisian cost revealed by mitochondrial COI sequences. Mol Biol Rep. 42:77–86. 25249225 10.1007/s11033-014-3743-z

[CIT0016] Miyamoto H, Machida RJ, Nishida S 2010 Genetic diversity and cryptic speciation of the deep sea chaetognath *Caecosagitta macrocephala* (Fowler, 1904). Deep-Sea Res Part II. 57:2211–2219.

[CIT0017] Øresland V 2000 Diel feeding of the chaetognath *Sagitta enflata* in the Zanzibar Channel, western Indian Ocean. Mar Ecol Progress. 193:117–123.

[CIT0018] Peijnenburg K, Haastrecht EKV, Fauvelot C 2005 Present day genetic composition suggests contrasting demographic histories of two dominant chaetognaths of the North-East Atlantic, *Sagitta elegans* and *S. setosa*. Mar Biol. 147:1279–1289.

[CIT0019] Peijnenburg KTCA, Fauvelot C, Breeuwer JAJ, Menken SBJ 2006 Spatial and temporal genetic structure of the planktonic *Sagitta setosa* (Chaetognatha) in European seas as revealed by mitochondrial and nuclear DNA markers. Mol Ecol. 15:3319–3338. 16968273 10.1111/j.1365-294X.2006.03002.x

[CIT0020] Pierrot-Bults AC 1982 Vertical distribution of Chaetognatha in the Central Northwest Atlantic near Bermuda. Biol Oceanogr. 2:31–61.

[CIT0021] Rabaoui L, Mejri R, Tlig-Zouari S, Bahri L, Hassine OKB, Tsigenopoulos CS 2011 Genetic variation among populations of the endangered fan mussel *Pinna nobilis* (Mollusca: Bivalvia) along the Tunisian Coastline. Hydrobiologia. 678:99–111.

[CIT0022] Telford MJ 2004 Evolution: affinity for arrow worms. Nature. 431:254–256. 15372015 10.1038/431254b

[CIT0023] Tse P, Hui SY, Wong CK 2007 Species composition and seasonal abundance of Chaetognatha in the subtropical coastal waters of Hong Kong. Estuarine Coastal Shelf Sci. 73:290–298.

